# Analyzing the Association between Hyperuricemia and Periodontitis: A Cross-Sectional Study Using KoGES HEXA Data

**DOI:** 10.3390/ijerph17134777

**Published:** 2020-07-02

**Authors:** Soo-Hwan Byun, Dae-Myoung Yoo, Jung-Woo Lee, Hyo-Geun Choi

**Affiliations:** 1 Department of Oral & Maxillofacial Surgery, Dentistry, Hallym University College of Medicine, Anyang 14068, Korea; purheit@daum.net; 2 Institute of Clinical Dentistry, Hallym University, Chuncheon 24252, Korea; 3 Hallym Data Science Laboratory, Hallym University College of Medicine, Anyang 14068, Korea; ydm1285@naver.com; 4 Department of Orthopedic Surgery, Hallym University College of Medicine, Anyang 14068, Korea; berrybear@hallym.or.kr; 5 Department of Otorhinolaryngology-Head & Neck Surgery, Hallym University College of Medicine, Dongtan 14068, Korea

**Keywords:** periodontitis, hyperuricemia, osteoporosis, periodontal-systemic disease interactions

## Abstract

Hyperuricemia arises from the buildup of excessive uric acid in the blood, and it is implicated in the development of periodontitis. The objective of this study was to investigate the association between hyperuricemia and periodontitis using a cross-sectional study design and Korean Genome and Epidemiology Study Health Examinee (KoGES HEXA) data. This prospective cohort study used epidemiological data from the KoGES from 2004 to 2016. Among 173,209 participants, 8809 with hyperuricemia and 126,465 controls (non-hyperuricemia) were selected. This study defined hyperuricemia as >7.0 mg/dL of uric acid in men and >6.0 mg/dL in women. This study analyzed the history of periodontitis among hyperuricemia and control participants. Participants’ age, gender, income, obesity, smoking, alcohol consumption, and nutritional intake were all examined. Chi-square tests, independent *t*-tests, and two-tailed analyses were used for statistical analysis. The adjusted OR (aOR) of hyperuricemia for periodontitis was 0.89 (95% confidence interval (CI) = 0.81–0.96, *p* = 0.005). This study demonstrated that hyperuricemia was associated with periodontitis. This finding meant that elevated uric acid levels could have a positive effect on periodontitis. However, further studies should be performed to determine the range of uric acid levels beneficial to periodontal health.

## 1. Introduction

Hyperuricemia arises when there is excessive uric acid in blood. It activates the crystallization of uric acid in joints and extraarticular areas [1]. Elevated uric acid levels can lead to several diseases, including a painful type of arthritis called gout [2,3]. Some population studies showed that approximately 15–20% of people are affected [1], and, in the United States, it is found in about 3.9% of the population [4]. The normal upper limit of uric acid level is 6.8 mg/dL, and a level above 7.7 mg/dL for men and 6.6 mg/dL for women is thought to be hyperuricemia [4].

Accelerated purine degradation and higher production or reduced excretion of uric acid could cause elevated uric acid levels [5]. They are also associated with health conditions such as cardiovascular disease, renal disease, metabolic syndrome, and diabetes [6,7]. Additionally, hyperuricemia could be related to osteolytic diseases such as osteoporosis [8]. At the normal human pH of 7.4, uric acid circulates and exists as an ionized form of urate. Urate production is activated by purine diets, high cell breakdown, and endogenous purine production [9]. Urate production is responsible for a minority of hyperuricemia cases, while urate excretion is responsible for hyperuricemia in 90% of the population [5]. Underexcretion is affected by reduced glomerular filtration, enhanced tubular absorption, and reduced tubular secretion [10]. The liver metabolizes purine, but it can also be made in any other organ, such as the intestines [11]. About two-thirds of uric acid is excreted through the kidney, and the remaining third is excreted into the intestine [12].

Most patients with hyperuricemia are asymptomatic and do not need long-term treatment. They usually have a history of consuming a purine-rich diet or an excess of beer [13]. Present medication and patient history should be checked to identify possible causes of hyperuricemia, such as higher production or insufficient renal excretion of urate. The most prevalent complaints of hyperuricemia patients are uric acid nephrolithiasis and gout [14].

Periodontitis is an inflammatory disease caused by periodontal pathogens that influence the periodontal tissue, including the gingiva and alveolar bone [15]. Dental plaque acts as a reservoir of bacterial pathogens, which can accelerate periodontitis. Pathogenic biofilm at the gingiva and tooth initiate the inflammatory processes in periodontal tissues, such as the production of chemokines and proinflammatory cytokines [16,17]. Inflammatory cytokines are the main regulators of inflammation in periodontitis [17]. The clinical symptoms of periodontitis are deep periodontal pockets, tooth loss, bone loss, and gingival bleeding. Periodontitis has a significant impact upon oral health and related quality of life. The mechanisms of periodontitis are related to oxidative stress, systemic diseases, and mitochondrial dysfunction [18,19]. Diabetes is one of the higher risk factors for periodontitis, and an interactional relationship between the two diseases has been reported [19]. Previous studies have shown that periodontitis can cause reactions in the immune system and a variety of diseases, including IgA nephropathy and glycosylated hemoglobin, leading to diabetes onset [20,21]. Isola et al. reported that patients with chronic periodontitis showed significantly lower serum levels of vitamin D compared to healthy controls [22]. The study showed that low serum vitamin D levels correlated with tooth loss and periodontitis, especially in chronic periodontitis patients. Evaluation of vitamin D levels should be recommended at the beginning of periodontal treatment, as it can predict and decrease the risk of chronic periodontitis aggravation [22,23].

Previous studies suggested that an association might exist between hyperuricemia and systemic diseases, including cardiovascular disease, periodontitis, and diabetes mellitus [8,24,25,26,27]. Few studies showed that increased uric acid levels could change the purine metabolism of oral diseases, including periodontitis and tooth resorption [28,29,30]. A relationship between increased uric acid levels and periodontitis has been demonstrated by both cross-sectional and interventional studies [28,31,32,33].

The objective of this study was to investigate the association between hyperuricemia and periodontitis using a cross-sectional study design and Korean Genome and Epidemiology Study Health Examinee (KoGES HEXA) data. It was hypothesized that elevated uric acid levels would increase the risk of periodontitis. In this study, patients with hyperuricemia were matched with control participants for age, gender, income, obesity, smoking, alcohol consumption, and nutritional intake.

## 2. Materials and Methods

### 2.1. Study Population and Data Collection

The ethics committee of Hallym University (20 February 2019) approved the use of these data. The requirement for written informed consent was waived by the Institutional Review Board. This prospective cohort study relied on data from the Korean Genome and Epidemiology Study (KoGES) from 2004 through 2016. KoGES is a large cohort study project in Korea that is designed to identify gene-environment factors and their interactions in common chronic diseases, such as type 2 diabetes, hypertension, metabolic syndrome, obesity, and cardiovascular disease. A detailed description of these data was described in a previous study [34]. Among the KoGES Consortium, we used KoGES health examinee (HEXA) data consisting of urban residence participants ≥ 40 years old. The HEXA data were from 2004 to 2013, and the follow-up data were from 2012 to 2016 and were obtained from the Korean Centers for Disease Control & Prevention (KCDC). KCDC selected the participants and performed the KoGES.

### 2.2. Participant Selection

**Figure 1:**
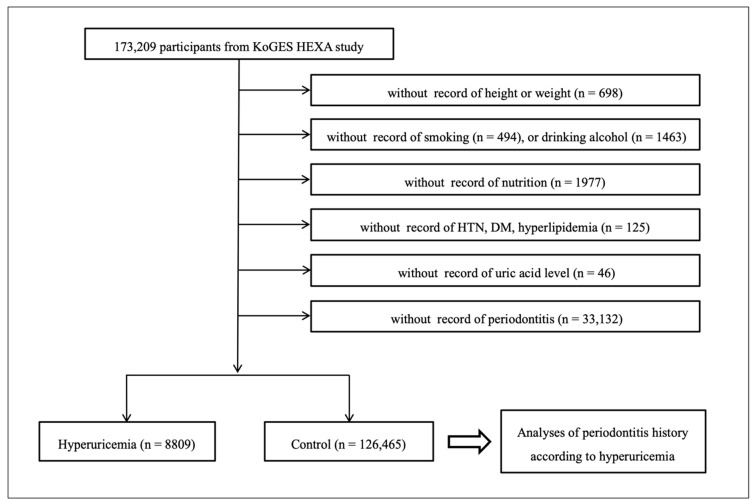
A schematic illustration of the participant selection process that was used in the present study. From 173,209 participants, 8809 with hyperuricemia and 126,465 controls (non-hyperuricemia) were selected.

Among 173,209 participants, we excluded the participants who lacked records of height or weight (*n* = 698), smoking history (*n* = 494), alcohol drinking habits (*n* = 1463), nutrition records (*n* = 1977), hypertension, diabetes mellitus, hyperlipidemia histories (*n* = 125), uric acid measurement (*n* = 46), and periodontitis history (*n* = 33,132). Many participants were excluded due to periodontitis histories because this was not surveyed from 2004 through 2006. Finally, 8809 patients with hyperuricemia and 126,465 patients without hyperuricemia (control participants) were selected (Figure 1). Then the study analyzed the histories of periodontitis between hyperuricemia and control participants.

### 2.3. Survey

The participants were asked about their previous history of hypertension, diabetes mellitus, hyperlipidemia, and periodontitis by trained interviewers. The questions were structured as follows: “Have you ever had a diagnosis of periodontitis?”, “Do you have any history of hypertension?”, and “Do you have any history of diabetes mellitus?”. All questions required a yes or no answer and the responses were confirmed by dentists and medical doctors. This study defined hyperuricemia as >7.0 mg/dL of uric acid in men and >6.0 mg/dL in women, following the previous studies [35,36]. The levels of uric acid were confirmed by blood analysis. Body mass index (BMI) was calculated by kg/m^2^ using the health checkup data. Smoking histories were categorized as non-smoker (<100 cigarettes in their entire life), past smoker (quit for more than one year), and current smoker. Alcohol drinking habits were categorized as non-drinker, past drinker, and current drinker. Their nutritional intake (total calories (kcal/day), protein (g/day), fat (g/day), and carbohydrate (g/day)) was surveyed by the food-frequency questionnaire validated by a previous study [37]. Income group was categorized as non-respondent, low income (<$2000 per month), middle income (~$2000–3999 per month), and high income (≥$4000 per month), using their household income.

### 2.4. Statistical Analyses

The Chi-square test was used to compare the rate of gender, income group, smoking, drinking alcohol, hypertension, diabetes mellitus, and hyperlipidemia histories. An independent *t*-test was used to compare the age, BMI, and nutritional intake.

To analyze the odds ratio (OR) of hyperuricemia for periodontitis, a logistic regression model was used. Crude and adjusted models (age, gender, income group, BMI, smoking, alcohol consumption, hypertension, diabetes mellitus, hyperlipidemia histories, and nutritional intake (total calories, protein, fat, and carbohydrate intake)) were used. In the subgroup analyses according to age, the dividing point was determined in the median age (≤52 years old and ≥53 years old).

Two-tailed analyses were conducted, and *p* values less than 0.05 indicated significance. The results were statistically analyzed using SPSS v. 24.0 (IBM, Armonk, NY, USA).

## 3. Results

The general characteristics of participants were different between the hyperuricemia and control groups (Table 1).

The adjusted OR (aOR) of hyperuricemia for periodontitis was 0.89 (95% confidence interval (CI) = 0.81–0.96, *p* = 0.005, Table 2). In the subgroup according to age and gender, the results were consistent only in young men. The aOR were 0.82 (95% CI = 0.69–0.98) in men aged ≤52 years, 0.77 (95% CI = 0.56–1.05) for women aged ≤52 years, 0.96 (95% CI = 0.84–1.09) in men aged ≥53 years, and 0.87 (95% CI = 0.73–1.03) in women aged ≥53 years.

In another subgroup analysis according to the past medical histories of hypertension, diabetes mellitus, and hyperlipidemia, the results showed statistical significance in the participants without hypertension, diabetes mellitus, and hyperlipidemia histories (Table 3).

## 4. Discussion

This study showed that the aOR of hyperuricemia for periodontitis was lower in all patients than that of the control group (Table 2). Periodontitis in hyperuricemia patients showed a significant association after adjustment for age, gender, income, smoking, alcohol consumption, hypertension, diabetes, hyperlipidemia, and nutritional intake (Table 1 and Table 2). This confirms our hypothesis for the study: that hyperuricemia might be a risk factor of periodontitis.

However, our results differed from those of most previous research. This means that the hyperuricemia could have a beneficial impact on periodontitis. In contrast, Banu et al. reported that an increased uric acid level was observed in periodontitis patients, as compared to non-periodontitis patients [28]. The study showed that uric acid could have a role in the inflammatory pathology of periodontitis.

Cao et al. also revealed that severe periodontitis was related to a higher uric acid level compared to mild/moderate periodontitis in patients with IgA nephropathy [31]. Babaei et al. demonstrated that non-surgical periodontal treatment exhibited a lowering effect on uric acid in periodontitis patients [33]. These findings indicated that an increased uric acid level was positively correlated with the severity of periodontitis [33].

Hyperuricemia shares various metabolic and inflammatory comorbidities with periodontitis, including diabetes, cardiovascular disease (CVD), osteoporosis, chronic kidney disease, and metabolic syndrome. Obesity and CVD are representative risk factors for both hyperuricemia and periodontitis. Previous studies have concluded that patients with obesity and CVD exhibit a higher risk of hyperuricemia and periodontitis [38,39,40,41,42,43]. Previous epidemiological studies reported a positive association between periodontitis and CVD [44,45]. A large cohort study and systematic review recently demonstrated a positive graded association between periodontitis and an increased risk of CVD [21,46]. Several studies have shown that risk factors, including age, smoking, and diabetes, are common to periodontal disease and atherosclerotic vascular disease. There has been an increase in the number of studies reporting that timely and regular periodontal treatment can prevent atherosclerotic vascular disease [47]. This study adjusted for obesity and hypertension by excluding the possibility of those metabolic comorbidities (Table 1). Another study concluded that an increased uric acid level would be a risk factor for periodontitis patients without adjustment for obesity and CVD [28]. The adjustment for various confounding factors is therefore a strength of this study, and it may explain the different results compared to the previous inconsistent studies. Osteoporosis is also a metabolic disease that could be related to both diseases, and it has been demonstrated to be a risk factor for periodontitis patients, especially among postmenopausal women [40,41,42,43,44]. On the contrary, Lin et al. found that elevated uric acid levels within the normal range were a positive factor for maintaining bone mineral density to resist bone resorption [45]. Veronese reported that hyperuricemia was independently associated with bone mineral density, and supported a protective role in bone metabolism disorders [48]. The antioxidative property of uric acid might induce the resistance in these studies. The results of the present study could be explained by a mechanism related to osteoporosis. Elevated uric acid could inhibit the risk of osteoporosis, and its reduced risk could be related to the lower aOR of hyperuricemia in periodontitis patients. Other factors, such as greater self-care in patients with hyperuricemia, could be responsible for the preventive effect on periodontitis. To our knowledge, only one study showed a similar finding that uric acid levels were significantly lower in the saliva of periodontitis patients than in healthy controls [49]. The study did not find the pathophysiology between periodontitis and uric acid levels. Further studies would be needed to explain a possible mechanism between periodontitis and elevated uric acid levels.

In spite of using large a population dataset, this study has some limitations. First, this study analyzed periodontitis in hyperuricemia and control participants according to their medical histories (Table 3). It showed statistical significance in the participants without hypertension, diabetes mellitus, and hyperlipidemia histories. However, since the number of participants with hypertension, diabetes, and hyperlipidemia was much smaller than the number without the diseases, the subsequent analysis may not be statistically robust. Second, it was impossible to calculate all the confounding factors for the association. Data from the KoGES did not have all of the potentially influencing factors. Medical treatment, drug intake, dental plaque existence, and plaque control could be confounders; however, they could not be included. Third, the data of the KoGES was designed based on a questionnaire survey. The survey used in this study could be inaccurate. The questionnaire asked the participants about their history of periodontitis diagnosis. The questions did not include the exact examination for gingivitis, gingival bleeding, or periodontitis. If the participants were checked by dentists or hygienists, the data would be more reliable. Fourth, gout and hyperuricemia are related to the metabolic diseases. This study considered the influence of metabolic diseases on the association between hyperuricemia and periodontitis, but the number of participants with these specific medical histories was too small to be analyzed properly (Table 3). This study also defined hyperuricemia as being above 7.0 mg/dL in men and above 6.0 mg/dL in women [35,36]. However, the definition of hyperuricemia varied in previous studies [4,35,36]. The results may have differed by diverse standards. Lastly, the reliability of the questionnaires for the frequencies of smoking, alcohol consumption, and nutritional intake could be unclear. To collect exact data, the reliability and validity of the questionnaire survey should be examined in future studies.

On the other hand, this study showed significant results with several advantages. To our knowledge, this study is the largest population-based study examining the association between hyperuricemia and periodontitis. Second, this study considered nutritional intake, age, gender, income, smoking, alcohol consumption, hypertension, diabetes, hyperlipidemia, and nutritional intake as influential factors in order to evaluate the independent association between hyperuricemia and periodontitis. Nutritional intake (including protein intake) and alcohol consumption (including beer intake) were further adjusted in this study. These factors would be essential adjustments for the plausible analysis of hyperuricemia as a systemic disease.

## 5. Conclusions

This study demonstrated that hyperuricemia was associated with periodontitis, and these preliminary findings suggest that elevated uric acid levels might have a positive effect on periodontitis. However, the study has a limitation in the possibility of incomplete data. In addition, excessive uric acid levels beyond the normal range were not analyzed independently; therefore, further studies should be performed to determine a more precise range of uric acid levels that would be beneficial to periodontal health.

## Figures and Tables

**Table 1:** General characteristics of participants.

Characteristics	Total Participants		*p*-Value
	Hyperuricemia	Control	
Age (mean, SD, year)	54.8 (7.9)	53.0 (8.3)	<0.001 *
Gender (*n*, %)			<0.001 *
Men	5949 (67.5)	41,298 (32.7)	
Women	2860 (32.5)	85,167 (67.3)	
BMI (mean, SD, kg/m^2^)	24.0 (2.9)	23.9 (2.9)	<0.001 *
Income (*n*, %)			0.086
Missing, no response	767 (8.7)	10,847 (8.6)	
Low	2562 (29.1)	36,456 (28.8)	
Middle	3352 (38.1)	49,739 (39.3)	
High	2128 (24.2)	29,423 (23.3)	
Smoking status (*n*, %)			<0.001 *
Non-smoker	4174 (47.4)	93,620 (74.0)	
Past smoker	2735 (31.0)	17,652 (14.0)	
Current smoker	1900 (21.6)	15,193 (12.0)	
Alcohol consumption (*n*, %)			<0.001 *
Non-drinker	2979 (33.8)	65,834 (52.1)	
Past drinker	505 (5.7)	4509 (3.6)	
Current drinker	5325 (60.4)	56,122 (44.4)	
Hypertension	3663 (41.6)	27,181 (21.5)	<0.001 *
Diabetes mellitus	986 (11.2)	10,003 (7.9)	<0.001 *
Hyperlipidemia	1695 (19.2)	17,996 (14.2)	<0.001 *
Nutritional intake			
Total calories (kcal/day)	1760.2 (580.6)	1749.4 (569.5)	0.070
Protein (g/day)	58.9 (26.6)	59.8 (26.4)	<0.002 *
Fat (g/day)	27.5 (18.5)	28.3 (18.2)	<0.001 *
Carbohydrate (g/day)	315.0 (95.2)	309.8 (92.8)	<0.001 *
Periodontitis (*n*, %)	657 (7.5)	9319 (7.4)	0.756

Independent *t*-test or Chi-square test. * Significance at *p* < 0.05.

**Table 2:** Crude and adjusted odds ratios (95% confidence interval) for periodontitis in hyperuricemia and control participants.

Characteristics	Odds Ratios for Periodontitis			
	Crude	*p*-Value	Adjusted ^†^	*p*-Value
Total participants (*n* = 135,274)				
Hyperuricemia	1.01 (0.93–1.10)	0.756	0.89 (0.81–0.96)	0.005 *
Control	1.00		1.00	
Age ≤ 52 years old, men (*n* = 21,504)				
Hyperuricemia	0.83 (0.70–0.98)	0.031 *	0.82 (0.69–0.98)	0.027 *
Control	1.00		1.00	
Age ≤ 52 years old, women (*n* = 45,454)				
Hyperuricemia	0.91 (0.66–1.24)	0.546	0.77 (0.56–1.05)	0.100
Control	1.00		1.00	
Age ≥ 53 years old, men (*n* = 25,743)				
Hyperuricemia	0.99 (0.87–1.13)	0.900	0.96 (0.84–1.09)	0.506
Control	1.00		1.00	
Age ≥ 53 years old, women (*n* = 42,573)				
Hyperuricemia	0.92 (0.78–1.09)	0.320	0.87 (0.73–1.03)	0.101
Control	1.00		1.00	

Logistic regression model, * significance at *p* < 0.05. ^†^ Models adjusted for age, gender, income group, BMI, smoking, alcohol consumption, hypertension, diabetes mellitus, hyperlipidemia histories, and nutritional intake (total calories, protein, fat, and carbohydrate intake).

**Table 3:** Crude and adjusted odds ratios (95% confidence interval) for periodontitis in hyperuricemia and control participants according to their past medical histories.

Characteristics	Odds Ratios for Periodontitis			
	Crude	*p*-Value	Adjusted ^†^	*p*-Value
With hypertension (*n* = 30,844)				
Hyperuricemia	1.02 (0.90–1.15)	0.773	0.97 (0.86–1.10)	0.613
Control	1.00		1.00	
Without hypertension (*n* = 104,430)				
Hyperuricemia	0.90 (0.80–1.01)	0.072	0.82 (0.73–0.92)	0.001 *
Control	1.00		1.00	
With diabetes mellitus (*n* = 10,989)				
Hyperuricemia	0.92 (0.74–1.15)	0.476	0.88 (0.70–1.09)	0.234
Control	1.00		1.00	
Without diabetes mellitus (*n* = 124,285)				
Hyperuricemia	1.01 (0.92–1.10)	0.850	0.89 (0.81–0.97)	0.010 *
Control	1.00		1.00	
With hyperlipidemia (*n* = 19,691)				
Hyperuricemia	0.92 (0.79–1.09)	0.342	0.87 (0.74–1.03)	0.112
Control	1.00		1.00	
Without hyperlipidemia (*n* = 115,583)				
Hyperuricemia	1.00 (0.91–1.10)	0.973	0.89 (0.80–0.98)	0.021 *
Control	1.00		1.00	

Logistic regression model, * significance at *p* < 0.05. ^†^ Models adjusted for age, gender, income group, BMI, smoking, alcohol consumption, hypertension, diabetes mellitus, hyperlipidemia histories, and nutritional intake (total calories, protein, fat, and carbohydrate intake).
